# Formation of therapeutic phage cocktail and endolysin to highly multi-drug resistant *Acinetobacter baumannii*: *in vitro* and* in vivo* study

**DOI:** 10.22038/IJBMS.2018.27307.6665

**Published:** 2018-11

**Authors:** Hayder Nsaif Jasim, Rand Riadh Hafidh, Ahmed Sahib Abdulamir

**Affiliations:** 1 College of Medicine, AL-Nahrain University, Medical Microbiology Department, Baghdad, Iraq; 2 College of Medicine, Baghdad University, Department of Microbiology, Baghdad, Iraq; 3 College of Medicine, AL-Nahrain University, Medical Microbiology Department, Baghdad, Iraq

**Keywords:** Acinetobacter baumannii, Bacteriophages, Drug resistance, Endolysin, Phage therapy

## Abstract

**Objective(s):**

Phage therapy is a potential alternative treatment for infections caused by *Acinetobacter baumannii*, a significant nosocomial pathogen, which has evolved resistance to almost all conventional antimicrobial drugs in poor hygiene and conflicts areas such as Iraq.

**Materials and Methods:**

Bacteriophages were isolated to highly resistant isolates of *A. baumannii* to form therapeutic phage cocktail, and to extract and evaluate native endolysin activity. Bacterial samples were collected in Al-Imamein Al-kadhimein Medical City Hospital. Phages were isolated from different regions in Baghdad city including (soil, sewage, irrigation channels). Phage endolysin was extracted from highly lytic phages that produced halo-like appearance around inhibition zone.

**Results:**

Up to 23 isolates of extensive- and pan- drug resistant (XDR, PDR) *A. baumannii *were isolated from patients with various infections, and 136 lytic phages specific to *A. baumannii *were isolated. Each bacterial isolate was sensitive to at least one lytic phage. Accordingly, a phage cocktail was formulated which remarkably minimized bacterial resistance to lysis by phages when compared to individual lytic phages. And, the phage cocktail succeeded in treating and saving life of all bacteremic mice with *A. baumannii *versus the non-treated group. In addition, the endolysin native activity to *A. baumannii* was evaluated in this study; endolysin revealed a potent antibacterial activity (> 1 log) reduction of bacterial density in just one hour of endolysin treatment.

**Conclusion:**

The phage therapy assessed in this study showed an ability to efficiently solve the problems of “superbug” bacteria by lysing effectively most XDR, PDR bacteria *in vitro* and *in vivo*. And, phage cocktail was shown to be superior over single-phage preparations in treating *A. baumannii* with much less resistance rate to therapeutic phages. Furthermore, intrinsic activity of native endolysin revealed promising results to tackling superbug pathogens.

## Introduction

Antibiotic resistance is an emerging global health disaster, resulting from the constant use and misuse of antibiotics in healthcare (1, 2). *Acenitobacter baumannii *is a Gram-negative, capsulated, opportunistic pathogen that is effortlessly spread in hospital intensive care units (ICU) (3). Most of *A. baumannii *clinical isolates are multi-drug resistant (MDR), extensively drug-resistant (XDR), and pan-drug resistant (PDR) bacteria, which greatly restricts the available treatment choices (4). To prevent returning to the dark “post antibiotics” era, there is an urgent need for new therapeutic agents against the MDR, XDR, PDR pathogens. To fight these bacteria, the scientists suggest a number of new therapeutics alternatives or complements to antibiotics against the “superbug” pathogens, of which *A. baumannii*. Interestingly, bacteriophage, or phage, therapy has been placed at the top of table presenting a possible alternative mean to tackle refractory bacterial infections (5). Phage therapy refers to the utilization of phages to treat bacterial diseases (6). Phages are very abundant in nature (7) and every bacterium is likely to have their own specific viruses that could be utilized as antibacterial agents (8-10). The host range of a given phage is often very specific to the sub-species level, which may confer an advantage over antibiotics by targeting pathogens without damaging commensal members of the host microbial community. However, this could be a drawback as it is not easy to find a phage for every pathogenic strain (11). Moreover, bacteria develop resistance to phages 10-fold easier than to chemical antibiotics (12). Therefore, using a mixture of lytic and specific phages to certain pathogenic bacteria would address the problems of narrow host range and anti-phage resistance (13). Hence, the formation of phage cocktail could save lives of uncountable patients suffering from serious and devastating *A. baumannii* infections resistant to the conventional antibiotics. This highlights the importance of using phage cocktails especially in a country like Iraq where *A. baumannii *flourishes in poor hygiene and areas of conflicts (14).Similar to phage cocktails, phage endolysins are lytic enzymes produced by bacteriophages during the last step of their replicative cycle. The enzymes degrade the cell wall of bacterial hosts and lead to cell lysis and phage progeny release (15). Endolysins are classified as a new class of antimicrobials for the treatment of drug-resistant bacterial infection because of their rapid action, low evidence of resistance development and low cytotoxicity against mammalian cells (15). In the case of Gram-negative bacteria, applications of specific endolysins are limited because the outer cell membrane (OM) prevents exogenously applied endolysins from attracting the peptidoglycan layer (15), Thus, many studies have focused on the enhancement of OM permeability using chelators such as EDTA (16), or by fusion of polycationic peptides to the Gram-negative endolysin facilitates outer membrane penetration allowing these new so-called Artilysin®s access to the Gram-negative peptidoglycan (17).

 Accordingly, the current study aims at testing the efficacy and biosafety of phage therapy, via using a single phage, and a phage cocktail to treat infections with MDR *A. baumannii* bacteria *in vitro *and *in vivo* and to extract anti-*A. baumannii* phage endolysins and determine their intrinsic lytic activity.

## Materials and Methods

***Specimen collection and identification***

Samples of bacteria were collected in Al-Imamein Al-kadhimein Medical City Hospital in Alkadymiya, Baghdad. Bacterial sampling was carried out during the period from September 2016 to November 2016. A total of twenty three, 23, different *A. baumannii* isolates (11 XDR, 12 PDR), belonging to hospitalized patients with various infections including septicemia, skin infection, severe UTI, pneumonia, and meningitis, were obtained from the central laboratory of the hospital. At the same day, samples were transferred to the laboratory of the Medical Microbiology Department in the College of Medicine, Al-Nahrain University to sub-culture bacteria on nutrient, MacConkey and blood agar then incubated at 37 ^⁰^C for 18-24 hr. Next day, all bacterial isolates were subjected to a full set of diagnosis including Gram staining, culture, and bio-chemical tests (18). Furthermore, the results of the identification of *A. baumannii *were confirmed by API 20E system.

***Antibiotic susceptibility test***

Antibiotic susceptibility test was carried out on *A. baumannii* isolates using Kirby-Bauer method (19). In this assay, 17 types of antibiotic disks were used as following imipenem (10 μg), ciprofloxacin (5 μg), colistin (10 μg), tigacyclin (15 μg ), gentamicin (10 μg), cefotaxime (30 μg), ceftazidime (30 μg), ceftriaxone (30 μg), trimethoprim/ sulphamethaxazole (1.25/23.75 μg), cefepime (30 μg), levofloxacin (10 μg), piperacillin(100 μg), tobramycin(10 μg), amikacin (30 μg), meropenem (10 μg), aztreonam (30 μg) and amoxicillin-clavulanicacid (20/10 μg). A 0.5 McFarland standards of bacteria was used and inoculated and spread by a sterile swab on Muller-Hinton agar Medium. Antibiotic discs were then placed on inoculated agar plates by forceps. The plates were left in incubator upside down at 37 ^⁰^C for 18-24 hr. 

***Bacteriophage sampling, isolation***

Different crude samples for phage isolation were obtained from different regions in Baghdad city including sewage, farm soil, feces of sheep, chicken litter, and swab from surgical lounge of several hospitals in Baghdad. Overnight bacterial broth (100 μl) was mixed with 2-3 ml of crude samplesand incubated overnight at 37 ^⁰^C until obtain specific lytic phage (21).

***Optimization and charachtarization of isolated phages***

Plaque characteristics were determined using top layer plaque assay and according to the following parameters: a) Diameter (mm) of the plaque. b) Shape of the plaque. c) Depth of the plaque. d) Margin cut. e) Clarity or turbidity of the plaque. Accordingly, the clearest and largest plaques were selected; moreover, small or turbid plaques were subjected to optimization by conducting serial passage in top layer plaque assays; at each run, the best of the best plaques, in terms of the above mentioned parameters, were selected in order to acquire better virulence characteristics of the isolated lytic phages. 

***Testing of bacterial resistance rate of A. baumannii to infecting bacteriophages***

The resistance rate of bacteria to infecting phages was measured. A piece from the bacterial lawn of the target bacteria equal in diameter to the phage lysis spot was cut by a sterile loop and put in 1.5 ml sterile Eppendorf tube containing one ml of normal saline to obtain the same number of bacteria that was present in the phage spot lysis zone.Then, the bacterial resistance rate was calculated as the following: 

Resistance rate=Number of resistant colonies per phage lysis spot/ number of bacterial colonies formed from the same size cut of bacterial lawn.

***Determination of the coverage rate of bacteriophage cocktails to A. baumannii***

In this approach, after mixing numerous phages in one suspension, randomly sampled 10 *A. baumannii *isolates were collected from patients in Al-Imamein Al-kadhimein Medical City Hospital. Ten (10) μl of the prepared bacteriophage cocktail suspension were spotted on to the surface of the overnight bacterial lawn and were allowed to dry before incubating at 37 ^⁰^C for 24 hr. On the next day, if a zone of lysis was developed at the spot where the phages suspension was applied, a susceptible bacterial isolate to phage cocktail was found. Then, the coverage rate of the formed bacteriophage cocktails was measured using this formula: 

Coverage rate=(number of bacterial isolates lysed by the phage cocktail / total number of bacterial isolates treated with the phage cocktail) X 100%.

***The assessment of the native activity of phage endolysin on A. baumannii bacteria extraction of endolysin***

About 100 ml of *A. baumannii *broth were incubated for 18-24 hr at 37 ^⁰^C. Next day, 250 ml of broth medium were added to the bacterial growth and incubated for another 3 hr at titer 1×10^9^ CFU/ml. Up to 10 ml of phage, titer 1×10^11^ PFU/ml (1:100 MOI), were mixed with bacteria for 20 min and were then put directly in ice and centrifuged at 10,000×g for 20 min and the sediment was collected. The sediment was suspended in 10 ml of 0.05 M phosphate buffer+5 mg deoxyribonuclease and incubated for 60 min at 37 ^⁰^C. EDTA(0.005 M) was added and centrifugation at 10,000×g for 1 hr and then the supernatant was taken. Disodium tetrathionate (0.3 M) was added and mixed for 1 hr at 4 ^⁰^C. Ammonium sulfate was added to 85% saturation and incubated for 18-24 hr at 4 ^⁰^C. Next day, centrifugation at 10,000g for 1 h and was resuspended in 5 ml of 0.05 M phosphate buffer saline (pH 7.5). Dialysis against 200 ml of the buffer at 4 ^⁰^C was conducted. The resultant solution was added to column chromatography sephadex G.100 in 0.1 M phosphate buffer saline pH 7.5, in 18×0.5 cm column. Each one ml of the resultant filtrate was collected in Eppendorf tube. From each Eppendorf tube, 10 μl of the filtrate were dropped by automatic pippete onto *A. baumannii *bacterial lawns of the specific bacteria to see which Eppendorf tube contains the lytic and native activity of endolysin. 

***Measurement of the native activity of endolysin on A. baumannii bacteria***

The endolysin activity was first checked by lysis on bacterial lawn and by decreasing the optical density of the bacterial broth when measured by a spectrophotometer. *A. baumannii *broth, composed of bacterial cells at mid-log phase OD(600=0.6), was centrifuged (4000×g, 30 min, 4 ^⁰^C) and then re-suspended in a phosphate-buffered saline (PBS) at pH 7.5. After assigning the tube that showed lysis in the bacterial lawn assay, 30 μl of this supposed-to-be endolysin-containing elute were added to 270 μl of the prepared bacterial broth at room temperature. Then, the optical density was measured spectrophotometrically every ten min for 1 hr at 600 nm (22). 

***In vivo*** **experiments of phage therapy on mice infected with highly resistant** ***A. baumannii***

In these experiments, white mice males of mean body weight 17±1.5 g and age 4-5 weeks were used. Mice were grouped into: mice injected with 0.3 ml of 10^8^ PFU/ml of a therapeutic phage alone (the phage group), 0.3 ml of 10^6^ CFU/ml of bacteria alone (the bacteremic group), 0.3 ml of 10^6^ CFU/ml of bacteria then, after 2 hr, with 0.3 ml of 10^8^ PFU/ml of a therapeutic phage (the test group) intreperitoneally (IP) in order to evaluate the phage side effects, bacterial virulence, and the efficacy of isolated and optimized phages to treat infected mice *in vivo*, respectively. In addition, a fourth group of mice that did not receive any kind of injections, the negative control group, was included. Besides, a mice group received 0.3 ml of 10^8^ PFU/ml of phage then after 2 weeks was treated as a test group in order to evaluate the *in vivo* phage therapy on immunized mice. For all these experiments, the physical activity of mice was monitored every day and the survival of mice was monitored for 9 days, or until the time of death. The health status of mice in each group was measured on a scale from 5 (normal) to 0 (death), based on the progressive status of bacteremia that is reflected by several clinical signs and physical activities (alertness, response to light or voice, feeding frequencies)(23).

## Results

***Characteristics of the isolates of Acinetobacter baumannii***

The isolates of *A. baumannii* came from patients infected with serious and life-threatening diseases including urinary tract infection, septicemia, wound infection, pneumonia, and meningitis. A total of 23 *A. baumanii *isolates were collected. The specimens were as follows: blood 7/23 (30.4%), urine 2/23 (9%), wound swab 7/23 (30.4%), diabetic foot 3/23 (13%), sputum 2/23 (9%), and C.S.F 2/23 (9%). The diseases, from which *A. baumanii* bacteria were isolated, were wound infection 10/23 (43.4%), urinary tract infection 2/23 (9%), septicemia 9/23 (39.1%), pneumonia 2/23 (9%), and meningitis 2/23 (9%). Hence, the most prevalent prevalent disease related to *A. baumanii *was wound infection followed by septicemia. 

The age of patients ranged from 1 day to 70 years and male to female ratio was 2.3.

***Antibiotic susceptibility test***

**Figure 1:**
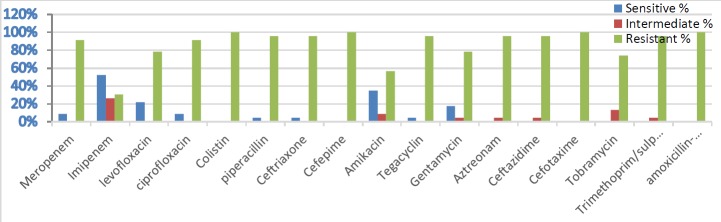
The rate of antibiotic sensitivity/resistance of 23 *Acinetobacter baumannii *isolates to a panel of 17 antibiotic disks commonly used in Iraq

The results showed that different *A. baumannii *isolates had different antibiotic sensitivity profiles; of 23 isolates included in the current study, 11 were XDR and 12 were PDR as shown in Figure 1.

***The characteristic features of the isolated and optimized phages***

The characteristics of the plaque assay of the isolated phages showed that plaques clarity ranged from clear, semi-clear, turbid, to semi-turbid, plaques size ranged from 0.5 to 6.5 mm, margin cut ranged from regular to irregular, and plaques shape from oval to circular. One hundred and thirty six (136) phages specific for 23 *A. baumannii* bacteria were isolated. Most of the isolated phages were highly lytic and produced obvious inhibition zone on target *A. baumannii* bacteria where plaque size was higher than 3 mm with full clarity of plaques; therefore, further optimization was not needed except for 25 phages which required further optimization in order to increase their lytic characteristics, (Table 1). 

The titer of the lytic phages isolated and optimized to *A. baumanii* were amplified and measured by using top layer plaque assay. Most phages reached high titers ranging between 10^8^-10^11^ PFU/ml using top layer plaque assay. 

**Figure 2:**
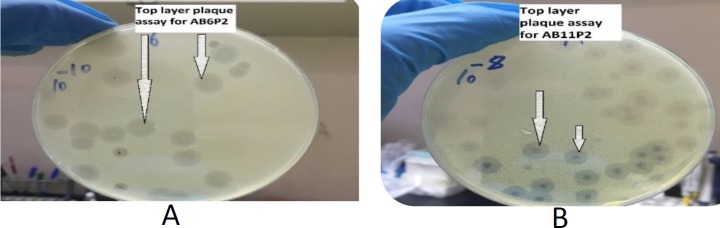
(A) Plaques produced by phage AB6P2 via top-layer plaque assay (B) plaques produced by phage AB11P2 via top-layer plaque assay

**Figure 3:**
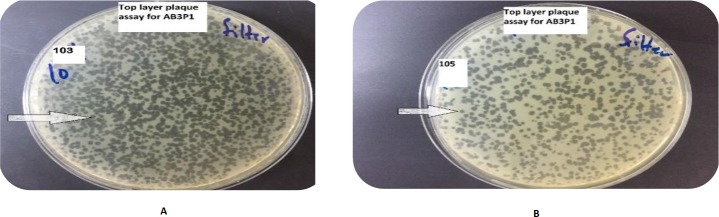
Top layer plaque assay for phage AB3P1 with different concentrations (A) AB3P1 with 103 PFU/ml (B) AB3P1 with 105 PFU/ml

The optimized specific and lytic phages were shown to be able to completely lyse the bacterial host in whatever manner of application of phages as demonstrated in Figures 2-3.

**Figure 4:**
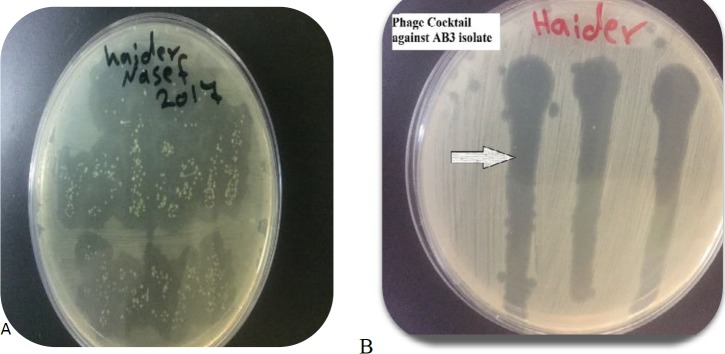
(A) Resistant *Acinetobacter baumannii* bacteria (AB3) to a single specific phage (AB3P1) (B) the phage cocktail completely lysed *A. baumannii* (AB3) without development of any resistant colonies

**Figure 5:**
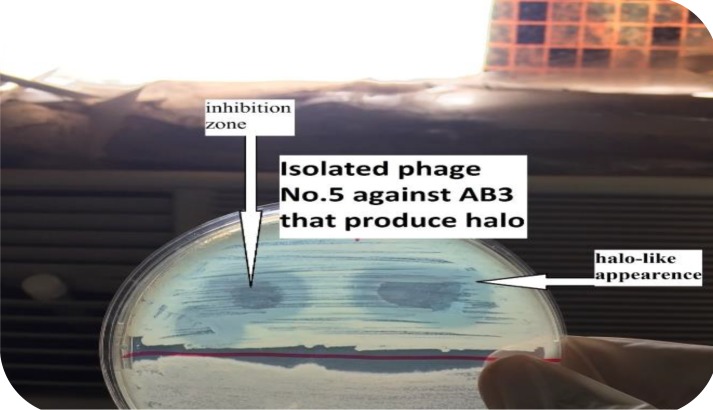
An obvious halo-like appearance around the inhibition zone produced by the lytic phage AB3P5 on the lawn of *Acinetobacter baumannii *isolate AB3

**Figure 6:**
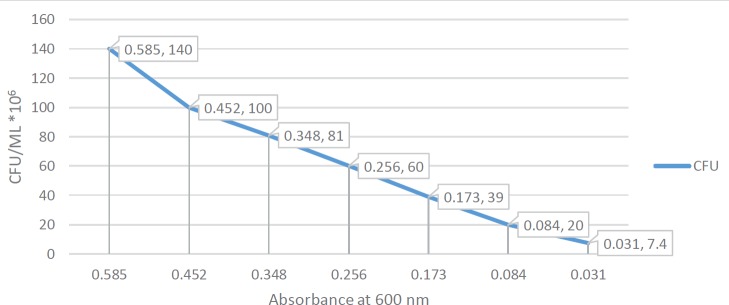
Interpolation of bacterial count in colony forming unit/ml with the optical density (600nm) of *Acinetobacter baumannii* broth treated with AB3 phage endolysin

**Table 1:** Morphological features of the isolated phages to *Acinetobacter baumannii* bacteria before and after optimization via top layer plaque assay

**Phage symbol**	**Plaque size (mm)**		**Plaque clarity**		**Plaque shape**		**Margin cut**	
	Before	After	Before	After	Before	After	Before	After
**AB1P1 **	0.5	1.5	Turbid	Semi-clear	Round	Round	Un-obvious	Regular
**AB1P2 **	0.3	1	Semi-turbid	Clear	Round	Round	Irregular	Irregular
**AB2P1 **	2	2.5	Semi-clear	Semi-clear	Round	Round	Regular	Regular
**AB3P1 **	0.8	1.5	Semi-turbid	Semi-clear	Oval	Oval	Irregular	Irregular
**AB3P2 **	0.5	0.5	Semi-clear	Clear	Oval	Oval	Regular	Regular
**AB3P3 **	2.5	4	Semi-Clear	Clear	Round	Round	Irregular	Irregular
**AB3P4 **	3.5	3.5	Semi-Clear	Clear	Oval	Oval	Irregular	Irregular
**AB4P1 **	3.5	7	Clear	Clear	Round	Round	Irregular	Irregular
**AB5P1 **	1	1.5	Turbid	Clear	Oval	Oval	Un-obvious	Irregular
**AB6P1 **	2	2	Semi-turbid	Semi-clear	Semi-round	Round	Irregular	Regular
**AB6P2 **	2	3.5	Semi-clear	Semi-clear	Oval	Oval	Irregular	Irregular
**AB9P1 **	0.5	0.5	Semi-Clear	Clear	Round	Round	Regular	Regular
**AB10P1 **	1.2	2.5	Clear	Clear	Round	Round	Regular	Regular
**AB10P2 **	1.9	5.5	Semi-turbid	Clear	Round	Round	Regular	Regular
**AB12P1 **	1	1	Semi-turbid	Semi-turbid	Oval	Oval	Irregular	Irregular
**AB15P1 **	3.5	5.5	Turbid	Clear	Round	Round	Un-obvious	Regular
**AB15P2 **	0.8	1	Semi-turbid	Clear	Round	Round	Regular	Regular
**AB17P1 **	1.7	3.5	Semi-Clear	Clear	Oval	Oval	Irregular	Irregular
**AB19P1 **	0.5	3	Semi-turbid	Semi-turbid	Round	Round	Un-obvious	Irregular
**AB19P2 **	0.8	1.5	Turbid	Clear	Round	Round	Un-obvious	Regular
**AB20P1 **	0.5	2	Semi-Clear	Clear	Oval	Oval	Regular	Regular
**AB21P1 **	1.5	6.5	Turbid	Semi-clear	Oval	Oval	Regular	Regular
**AB21P2 **	1	2	Semi-turbid	Semi-clear	Round	Round	Irregular	Irregular
**AB22P1 **	0.5	1.5	Turbid	Turbid	Oval	Oval	Un-obvious	Regular
**AB22P2 **	2.3	4.5	Semi-turbid	Semi-turbid	Oval	Oval	Irregular	Irregular

**Table 2:** The biokinetic parameters of optimized phages: Infective percentage (IP %), Burst time (BT) in min, and Burst size (BS) in number of progenies of the randomly selected bacteriophages to Acinetobacter baumannii bacteria

	IP %	BT	BS
AB1P2	91.3	40	170
AB3P4	94.5	45	245
AB5P1	76.4	35	220
AB7P3	85.2	30	160
AB9P1	88	45	210
AB10P2	86.6	45	145
AB14P1	77.5	40	190
AB15P3	82	30	200
AB17P2	80	40	130
AB20P1	79	35	185

**Table (3):** The health score of the test group of mice injected IP with AB3 bacteria and AB3P phage in comparison with the bacteremic group injected only with AB3 bacteria

**Hour**	**Health scores** **Bacteremic group **	**Median** **of health scores of acteremic group**	**Health scores ** **of test group **	**Median of health** **scores of test group**	*P*-value** (Mann** **Whitney test)**
**0.5**	4,4,5,5,5	5	4,4,4,5,5	4	0.84
**1**	4,4,4,4,3	4	4,4,4,4,5	4	0.64
**1.5**	3,3,3,4,4	3	3,3,4,3,4	3	0.76
**2**	3,2,3,3,3	3	3,3,3,4,3	3	0.64
**2.5**	3,2,2,2,2	2	4,3,4,4,4	4	0.04
**3**	2,1,2,1,1	1	5,4,5,5,5	5	>0.01
**3.5**	1,0,1,0,0	0	5,5,5,5,5	5	>0.01
**4**	0,0,0,0,0	0	5,5,5,5,5	5	>0.01

**Table (4):** Health score of the phage group injected IP with AB3-specific phage alone in comparison with the negative control group of mice

**weeks**	**Health scores** **of phage group**	**Median of health** **scores of** **phage group**	**Health scores of** **negative control group**	**Median of health** **scores ** **of** **negative control group**	*P*-value** (Mann** **Whitney test)**
**1 **	5,5,5,4,5	5	5,5,5,5,5	5	1
**2**	5,5,5,5,5	5	5,5,5,5,5	5	1
**3**	5,5,5,5,5	5	5,5,5,5,5	5	1
**4**	5,4,5,5,5	5	5,5,5,5,5	5	1
**5**	5,5,5,5,4	5	5,5,5,5,5	5	1

**Table (5):** Health score of AB3 non-immunized and AB3 immunized mice groups treated with AB3-specific phage

**Hour**	**Health scores of** **immunized mice treated with phage**	**Median of health ** **scores of immunized mice treated with phage**	**Health scores of non-immunized mice treated with phage **	**Median of health scores of non-immunized mice treated with phage**	*P*-value** (Mann** **Whitney test)**
**0.5**	**4,4,5,5,5**	**5**	**4,4,4,5,5**	**4 **	**0.84**
**1**	**4,3,4,4,4**	**4**	**4,4,4,4,5 **	**4 **	**0.64**
**1.5**	**3,3,3,4,4**	**3**	**3,3,4,3,4 **	**3 **	**0.76**
**2**	**3,3,3,3,3**	**3**	**3,3,3,4,3 **	**3 **	**0.8**
**2.5**	**4,4,4,4,4**	**4**	**4,3,4,4,4 **	**4 **	**1**
**3**	**4,4,5,4,5**	**4**	**5,4,5,5,5 **	**5 **	**0.52**
**3.5**	**5,5,5,4,5**	**5**	**5,5,5,5,5 **	**5 **	**1**
**4**	**5,5,5,4,5**	**5**	**5,5,5,5,5 **	**5 **	**1**

***The characteristics of the isolated and optimized phages in terms of biokinetic assay***

In the current study, 10 bacteriophages to differentbacterial isolates were randomly selected to give representative values of biokinetic characteristics. The results in this study showed that the average burst time (BT) was 73.5 min ranging between 30 to 45 min. The maximum burst size (BS) was 245 progeny, while the minimum BS was 130 progeny and the average BS was 187.5 progeny. The average infective percentage (IP %) was 85.45% ranging between 74.4% and 94.5%, as shown in Table 2.

***Formation of phage cocktail to A. baumannii***

A phage cocktail was formed by mixing 64 phages specific for 23 *A. baumannii *isolates (AB1-AB23). All bacterial isolates, except AB2 and AB8, were targeted by more than one phage; the most targeted isolate was AB3 where 6 different phages shared the same specificity towards this isolate.

***Bacterial resistance to a single phage versus phage cocktail***

Up to 18/23 (78.3%) of *A. baumannii* bacteria were completely sensitive to the applied lytic phages with zero resistant bacterial colonies. So, only 5 out of 23 isolates (21.7%) of *A. baumannii* were shown to develop some level of resistant colonies in the inhibition zone at the spot of lytic phage application. On the other hand, the formed phage cocktail was shown to remarkably minimize the number of the resistant bacterial colonies appeared to individual phages. The results revealed that once* A. baumannii* isolate develops resistance to a one

member of the phage cocktail, this bacterial isolate was still sensitive to other phage members in the same cocktail as shown in Figure 4.

***The coverage rate of the formed phage cocktail to A. baumannii bacteria***

Ten (10) *A. baumannii *isolates were collected randomly after the formation of the phage cocktail. The collected specimens were not biased towards particular disease, site of infection, or patients’ age or sex. The formed phage cocktail was able to form a clear inhibition zone on the most tested bacterial lawns. The coverage rate of the formed phage cocktail was calculated. The phage cocktail was shown to be able to lyse 7/10 (70%) of *A. baumannii* and thus the coverage rate was 70%.

***Determination of the native activity of phage endolysin on A. baumanii***

Some phages were found to produce a halo-like appearance around the inhibition zone produced by some lytic phages as shown in Figure 5. This halo-like appearance suggested a native endolysin production from phage. A specific phage endolysin to *A. baumannii* was extracted successfully by using sephadex G100 column chromatography. The Eppendorf tube number two showed positive results for phage endolysin. The optical density of *A. baumannii* broth was measured initially at zero time, just before the addition of the corresponding endolysin, then it was measured every 10 min up to 1 hr and it showed obvious decline in optical density of bacterial broth with time. According to t-distribution test, there was a significant difference between the test groups, bacteria treated with endolysin and control group, bacteria alone with PBS, (*P*=0.00134). Moreover, the overall enzymatic activity of extracted native endolysin was quantified by turbidometric reduction analysis, 270 μl of exponentially growing *A. baumannii* (AB3) cultures (1.4 x 10^8^ CFU/ml) were challenged to 30 μl of extracted native endolysin at room temperature. A. baumannii optical density and viability counts were reduced from 0.585 to 0.031 after one hour of treatment, compared with the untreated control group that continued to grow (from 577 to 624 after one hour). It was shown to be 0.0092 ΔOD/min. By using standard curve measurements to interpolate OD values to bacterial count, it was shown that the endolysin native activity surpassed 1.4 log reduction threshold after one hour of treatment as shown in Figure 6.

***In vivo experiments of phage therapy***

The results in the current study showed that the test group, which was injected IP with AB3 isolate and in 2 hr was treated IP with AB3-specific phages, stayed alive for more than 6 weeks, with full physical activity. By using Mann Whitney test, there was no significant difference in the median of health score between the bacteremic (without phage therapy) and test (with phage therapy) groups (*P*=0.64) during the 2 hr period after the bacterial injection and before the phage injection, Table 3. On the other hand, 2.5 to 4 hr after phage administration, the median of health score of the test group was far higher than the bacteremic group (*P*< 0.01), Table 3. Therefore, 4 hr of bacteremia with AB3 *A. baumannii* were enough to kill the bacteremic mice while mice treated with phage 2 hr after the start of bacteremia were 100% saved and survived in healthy condition as shown in Table (3). Referring to the phage group, that was injected IP with AB3-specific phage at concentration 10^8^ PFU/ml alone, mice stayed alive, without any change in their health score (*P*-value =1) as shown in Table (4). More to the *in vivo* experiment, there was no significant difference in the median of the health score between AB3-specific phage immunized versus non-immunized groups of mice (*P*>0.05).

## Discussion

All of the isolates of *A. baumannii *were shown to be completely resistant to several antibiotics as follows:

Cefepime, Cefotaxime, colistin, and amoxicillin-clavulanic acid. However, the resistance rate to other antibiotics was less than 100% and ranged from 95.65% to 30.43%. The multiple drug resistant status reported in current study is in agreement with the findings of other recent studies carried in Iraq (24, 25)but this study disagrees with a study carried out in USA which reported that approximately 50% of patients are with colistin-resistant *A. baumannii* (26). The variation in the results may be due to differences in the time when the studies were conducted, or simply differences in the geographic areas. The complete resistance of *A. baumannii* isolates collected in this study to colistin might be attributed to the major mechanism of colistin resistance in *A. baumannii*, namely modification of lipopolysaccharide (LPS) outer membrane via adding phosphor ethanol amine to the hepta -acylated lipid A structure (27-30). In this regard, phage therapy could offer one of the best applicable solutions to overwhelm the problem of antibiotics resistance of bacteria in Iraq, the Middle East, and in the world (31). One of the striking merits of using phages over antibiotics in a country like Iraq is that phages are self-amplifying in the site of infection; so, phages can be given to patients in a single dose. Hence, incompliance of patients will not affect the success of the course of therapy. In this study, the lytic and specific phages to *A. baumannii *were isolated from various environmental sources; the main source was sewage; this finding is in line with other studies (32, 33). Another main source of phages in this study was waste water (34). The current study revealed that sewage was the best source to isolate highly lytic and specific phages to *A. baumannii* (35). 

The current study showed successful *in vitro* use of both single phage and phage cocktail to lyse *A. baumannii* XDR or PDR isolates. Nevertheless, this study revealed a superiority of the phage cocktail over the single phage in lysing *A. baumannii* bacteria without development of resistant colonies to phage therapy. Consequently, such phage cocktails are good candidates to prevent the emergence of phage-resistant mutants (21, 36). The results of the current study revealed that using phage cocktails provides several advantages. Firstly, phage cocktails broaden the strain-specific range of infective phages. This permits effective therapy of a broader spectrum of bacteria within the same *A. baumannii *species (37, 13). Secondly, phage cocktails solve the serious obstacle of the development of *A. baumannii *resistance to attacking phages. It was stated that using phage cocktails is the finest choice for effective phage therapy without suspicions of rapid emergence of bacterial resistance (38). Therefore, the phage cocktail used in this study ensured these two important goals. 

Each *A. baumannii *isolate have more than one receptor and each receptor is recognized by a different phage to attach and invade (13). This explains why each bacterial isolate was invaded by more than one different phage. Therefore, when a bacterial isolate develops resistance to one phage in the phage cocktail, it is still sensitive to other phages in the same phage cocktail. From the findings of the top layer plaque assay, each member of the phage cocktail was unique, and from the findings of the bacterial resistance rate to single phage versus phage cocktail, the phages used in this study seem to target different receptors on the cell wall of *A. baumannii*. This provides evidence on the superiority of using phage cocktails. 

 The coverage rate of the formed phage cocktail in this study was shown to be very high, up to 70%. Such high coverage paves the road to successful and ready-to-use therapy of serious and life-threatening infections of *A. baumannii. *Nevertheless, in this study, it was proven that in few months, a phage cocktail of 64 anti-*A. baumannii *specific phages was formed. The formed phage cocktail could save lives of uncountable patients suffering from serious and devastating *A. baumannii *infections resistant to the conventional antibiotics. This highlights the importance of using phage cocktails especially in a country like Iraq where* A. baumannii *flourishes in poor hygiene and areas of conflicts (39).

 The native activity of endolysin shown in the current study is in harmony with few studies examined the native activity of endolysin from phages infect Gram-negative bacteria such as* A. baumannii, Pseudomonas aeruginosa* and *Escherichia coli (*40-45). The current study highlights the intrinsic antimicrobial activity of endolysin produced from phages against Gram-negative bacterial pathogens. Native endolysin activity is a good candidate for the therapeutic/disinfectant endeavor to control nosocomial infections caused by MDR bacteria, particularly MDR *A. baumannii (*40). The intrinsic antibacterial activity of endolysin against Gram-negative needs the ability of endolysin to get through the outer membrane of these bacteria. This might explain why endolysins from phages infecting Gram-negative hosts are mostly small single-domain globular proteins (molecular mass between 15 and 20 kDa), and usually without a specific CBD module (41). These lysins likely better fulfill the catalytic role of classical enzymes (aiding multiple catalytic reactions during cell lysis), as opposed to their Gram-positive counterparts, which are proposed to bind to one site and have a very low off-rate (46-49). 

Referring to the *in vivo* results, the current study highlighted several points to discuss. First, we selected one of the most pathogenic bacteria to mice, which have the capability to kill mice within only 3.5-4 hr. Second, the therapeutic phage used in this study, AB3-specific phage, stopped and reversed the worsening health score of mice and succeeded in eradicating the morbidly effects of the septicemic state of *A. baumannii *AB3. Even better, the therapeutic phage succeeded in rescuing life of the bacteremic mice group. This was a definitive proof on the efficacy and eligibility of using specific, optimized, highly lytic phages in treating deadly *A. baumannii* infections. These findings are in harmony with findings of several previous reports (50, 51). Third, the findings of this study revealed solid proof on the safety of therapeutic phages. The experimental mice group that was injected IP with 10^8^ PFU/ml of therapeutic phage alone showed no change in health score and physical activity when compared to the negative control group. Moreover, mice were clear from any side effect for more than 4 weeks. This outcome is in line with other studies conducted to evaluate the safety of therapeutic phages *in vivo (*52, 53). The safety of bacteriophages is attributed to the fact that phages attach and react to receptors only found on prokaryotic cells. So, phages are incapable to invade eukaryotic cells and cause infections because no receptors specific for phage are on the eukaryotic cells (15).

Referring to *in vivo *phage therapy to immunized and non-immunized mice groups, the group of mice previously exposed to therapeutic phage (immunized) was unable to mount an effective immune response to the same phage at the second encounter. This might be explained by several points of view. First, bacteriophages are hyper-available in our environment, and plentifully found in what we eat and drink (54), so, our bodies in general and our immune system in particular get used to the existence of phages and no violent and aggressive immune reaction occur against phages (55). Second, if immune response was mounted, it is evident that it was not enough to hinder therapeutic phage from reaching and lysing *A. baumannii.* Third, host bacteria act as a shelter to attacking phages from the hostile environment of immune system; therefore, the higher the extent of bacterial infection, the more shelter provided by host bacteria to the attacking phages and vice versa. Fourth, the phage kinetics, as shown in the current study with burst size up to 150 and infection percentage up to 94%, are far more efficient than that of bacteria leading to an effective eradication of bacterial pathogen rapidly before the immune response become capable of wiping out the attacking phages. Therefore, the immune response produced by mice did not hinder the efficacy of phage therapy, these results are in line with previous reports (55, 56). 

## Conclusion

 Taken together, the findings of this study indicate that *A. baumannii *in Iraq are mostly XDR and PDR bacteria; such abnormally high rate of multiple drug resistance necessities novel methods to tackle this impeding health risk on community. Therefore, phage therapy assessed in this study was shown to be able to solve the problem of superbug bacteria by lysing effectively most XDR and PDR bacteria *in vitro*. And, phage cocktails were shown to be superior over single-phage preparations in treating *A. baumannii* with much less rate of resistance to therapeutic phages. In addition, the native activity of endolysin from phages specific to *A. baumannii* revealed a potent antibacterial activity, >1 log reduction of bacterial density in just 1 hr of endolysin treatment; this provided evidence to tackle Gram negative bacteria by using low molecular weight endolysins which are of high level of native antibacterial activity. The results from *in vivo* experiments provide a solid proof that the formed phage cocktail as well as the single phage preparations are highly effective in treating life-threatening bacteremic cases and can quickly restore health condition back to normal; moreover, phage therapy was shown to be safe when given systemically without any side effects.
